# Evolutionary Genetics of an *S*-Like Polymorphism in Papaveraceae with Putative Function in Self-Incompatibility

**DOI:** 10.1371/journal.pone.0023635

**Published:** 2011-08-31

**Authors:** Timothy Paape, Takashi Miyake, Naoki Takebayashi, Diana Wolf, Joshua R. Kohn

**Affiliations:** 1 College of Biological Sciences, University of Minnesota, St. Paul, Minnesota, United States of America; 2 Institute of Arctic Biology, University of Alaska, Fairbanks, Alaska, United States of America; 3 Department of Biology and Wildlife, University of Alaska, Fairbanks, Alaska, United States of America; 4 Department of Biology, University of California San Diego, La Jolla, California, United States of America; Ecole Normale Superieure, France

## Abstract

**Background:**

*Papaver rhoeas* possesses a gametophytic self-incompatibility (SI) system not homologous to any other SI mechanism characterized at the molecular level. Four previously published full length stigmatic *S*-alleles from the genus *Papaver* exhibited remarkable sequence divergence, but these studies failed to amplify additional *S*-alleles despite crossing evidence for more than 60 *S*-alleles in *Papaver rhoeas* alone.

**Methodology/Principal Findings:**

Using RT-PCR we identified 87 unique putative stigmatic *S*-allele sequences from the Papaveraceae *Argemone munita*, *Papaver mcconnellii*, *P. nudicuale*, *Platystemon californicus* and *Romneya coulteri*. Hand pollinations among two full-sib families of both *A. munita* and *P. californicus* indicate a strong correlation between the putative *S*-genotype and observed incompatibility phenotype. However, we also found more than two *S*-like sequences in some individuals of *A. munita* and *P. californicus*, with two products co-segregating in both full-sib families of *P. californicus*. Pairwise sequence divergence estimates within and among taxa show *Papaver* stigmatic *S*-alleles to be the most variable with lower divergence among putative S-alleles from other Papaveraceae. Genealogical analysis indicates little shared ancestral polymorphism among *S*-like sequences from different genera. Lack of shared ancestral polymorphism could be due to long divergence times among genera studied, reduced levels of balancing selection if some or all *S*-like sequences do not function in incompatibility, population bottlenecks, or different levels of recombination among taxa. Preliminary estimates of positive selection find many sites under selective constraint with a few undergoing positive selection, suggesting that self-recognition may depend on amino acid substitutions at only a few sites.

**Conclusions/Significance:**

Because of the strong correlation between genotype and SI phenotype, sequences reported here represent either functional stylar *S*-alleles, tightly linked paralogs of the S-locus or a combination of both. The considerable complexity revealed in this study shows we have much to learn about the evolutionary dynamics of self-incompatibility systems.

## Introduction

Angiosperms possess various genetic mechanisms to reduce inbreeding by rejecting self-pollen. Single locus gametophytic self-incompatibility (GSI) occurs in several plant families [1], [2] and is defined by rejection of haploid pollen in the stigma or style if the pollen carries a self-incompatibility allele (*S*-allele) that matches either allele of the ovule parent. Crossing studies [3] of self-incompatible *Papaver rhoeas* estimated that this species maintains 66 *S*-alleles at the gametophytic *S*-locus [4]. Molecular studies led to the cloning of two stigmatic *S*-alleles [5], but despite considerable efforts to clone additional haplotypes, Kurup *et al.* [6] were only able to sequence one additional *S*-allele from *P. rhoeas* (*S8*) and one from the congener *P. nudicaule* (*Sn1*). It is thought that the very high level of divergence among *Papaver S*-proteins confounded the isolation of more *S*-alleles by standard PCR and hybridization techniques [6]. Sequence analysis of these alleles and studies characterizing the molecular mechanism of pollen tube inhibition revealed that this system bears no homology to the *S*-RNase-based gametophytic self-incompatibility system found in the Solanaceae, Plantaginaceae and Rosaceae, or the sporophytic SI system (SSI) found in Brassicaceae where the principle stylar product of the *S*-locus is an *S*-receptor kinase (*SRK*; reviewed in [7], [8]).

The *S*-locus products secreted in the pistil of *Papaver* are small proteins (∼15 kDa) that bind to corresponding pollen *S*-receptors when haplotypes match, initiating receptor mediated programmed cell death [9]–[12]. The *Papaver* stigmatic *S*-proteins possess four conserved cysteine residues and the predicted secondary structure is comprised of ß-strand motifs linked by seven hydrophilic loops [13], [14]. Other types of molecular recognition proteins [15]–[17] have distinct hypervariable regions interrupted by conserved regions contrasting with *Papaver* stigmatic *S*-proteins where variability is at the 5′ end (∼30 amino acids) and at scattered residues throughout the sequence. In *Papaver* stigmatic *S*-proteins display 40–50% amino acid divergence between pairs of alleles, as do the recently-discovered pollen *S*-proteins they interact with [12]. An early scan of the *Arabidopsis* genome revealed as many as 100 ‘*S*-protein homologues’ (SPHs; [18]) and another SPH was described in tomato [19]. SPHs are the closest known homologs of the *Papaver* stigmatic *S*-protens but no function has yet been ascribed to any SPH.

The *S*-RNase and *SRK* systems both exhibit extensive ancestral polymorphism shared across genera [16], [20], [21]. This reflects balancing selection preserving variation at the self-incompatibility (*S*-) locus for millions of years [22], [23] and makes it possible to infer demographic events that occurred before the origin of extant species [21], [24]–[26]. The extent of shared ancestral *S*-locus polymorphism in the Papaveraceae is unknown because, while many other Papaveraceae are self-incompatible [27]–[30], *S*-locus sequence polymorphism has not been investigated outside of *Papaver* [6].

Here we attempt to isolate homologous *S*-alleles from SI species of Papaveraceae, focusing primarily on wild relatives of *Papaver rhoeas*. Three species native to California are investigated: *Argemone munita*, *Platystemon californicus*, and *Romneya coulteri*. Both *A. munita* and *R. coulteri* are tetraploid and while the former typically grows as a desert annual, the latter is a perennial and often grows clonally. *Platystemon californicus* is a diploid summer annual and the only member of its genus. Self-incompatibility reactions of all three native Californian species have been examined using aniline blue staining and other microscopy techniques [31]. In all cases self pollen tubes arrest immediately upon contact with living female tissue, as is found in *Papaver*. We also look for *S*-like polymorphism in two Alaskan species: *Papaver mcconnellii*, a mostly tetraploid perennial that grows on rocky scree in the Yukon Territory [32] and *P. nudicaule*, an annual or short-lived perennial that may be diploid or tetraploid. Our goals in this study were to: a) amplify putative *S*-alleles from these species based on known *Papaver S*-allele sequences, b) determine whether putative *S*-haplotypes predict the SI phenotype in greenhouse crosses, c) assess genealogical characteristics and patterns of sequence variation in putative *S*-alleles within and among species.

## Results

For most taxa studied, stigmatic sequences of putative *S*-alleles were amplified from stigmatic tissue using RT-PCR althought some sequences from *Papaver* spp. were amplified from genomic DNA (see methods). Sequence data from have been deposited with the EMBL/GenBank Data Libraries under accession nos. (GQ351602–GQ351691).

**Figure 1:**
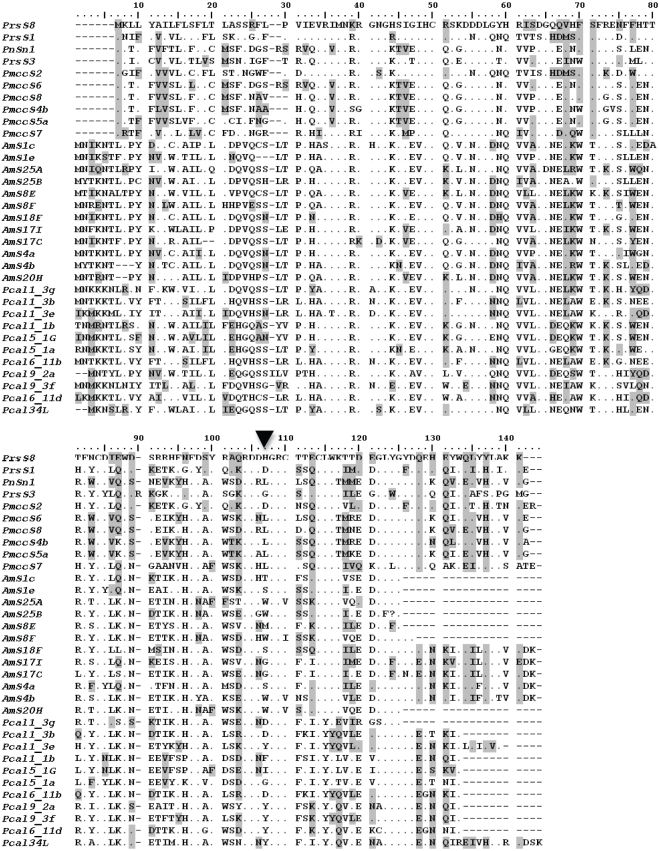
Amino acid alignment of all sequences containing 5′ ends. All ten *Papaver* spp. sequences shown are full length possessing both 5′and 3′ ends. Dots are invariable amino acids, gray indicates 40% shared identity threshold. Black arrow indicates the amino acid (site 107) used in the site directed mutagenesis experiment ([14]; see text] which is predicted to be under positive selection by both PAML [73] and OmegaMap [34] analyses.

The sequences amplified were highly polymorphic as expected for putative *S*-alleles and showed the nearest match in NCBI nucleotide tBLASTx queries to the published *S*-alleles of *Papaver*. Thirty-seven complete or partial sequences were amplified from *A. munita*. We amplified two sequences from 12 *A. munita* individuals, only one sequence from 10, while in one plant three sequences were found. A total of 31 partial *P. californicus* sequences were obtained using 3′ RACE and nested PCR and eleven of these sequences were completed using 5′ RACE (Figure 1). For *P. californicus*, two products were found for 11 individuals, only one product was found for 10 individuals and three or four products were found for 4 individuals. Two sequences were also found for all 12 *Romneya coulteri* sampled for a total of six unique sequences. PCR from stigmatic tissue resulted in six new full-length putative *S*-alleles from *P. mcconnellii* and one from *P. nudicaule*. For *Papaver* species, only one allele was detected for each of the seven individuals tested.

### Hand pollinations —*Argemone munita*

Two *A. munita* full-sib families were genotyped (‘8-1’ and ‘25-4’) using allele specific primers for the 5′ ends of putative *S*-allele sequences. Family 8-1 was generated by crossing plant 8 (with alleles *S8a* and *S8b*) with plant 1 (with alleles *S1c* and *S1d*), while family 25-4 was generated by crossing plant 25 (*S25a* and *S25b*) with 4 (*S4a* and *S4d*). Here, we denote putative *S*-alleles with plant individual ID (digits; e.g., 4) and a single letter (e.g., ‘a’, ‘c’ or ‘d’) indicating different sequences amplified from the same individual. Sequences segregated in an allelic fashion in both F1 families (Table S1). When crossing full-sibs, we expect that crosses among plants with only one shared allele (predicted semi-compatible) will set fruit, whereas crosses between matching genotypes (predicted incompatible) will not. As expected, 100% of the 64 pollinations among individuals possessing only one shared allele produced fruit, while none of 15 matching-genotype crosses from family 8-1 set fruit (Table 1). However, in family 25-4, some matching-genotype crosses set fruit while others did not (Table 1). Unexpected fruit set only occurred in crosses among plants that had the sequence *S25b*. We recorded 59% fruit set in 34 matching-genotype pollinations involving putative allele *S25b* and zero fruit set in 43 pollinations not involving *S25b* (Table 1). This could suggest that *S25b* is a weak or non-functional *S*-allele or that the *S25b* sequence is not linked to the functional *S*-locus. Overall, this set of crossing data supports that at least seven out of eight putative *S*-alleles that segregated as heterozygous products (*S25a, S4a, S4d, S8a, S8b, S1c, S1d*) are at least linked to functional *S*-alleles.

**Table 1:** Fruit set following hand pollinations among full sibs of *Argemone munita* with matching genotypes (predicted incompatible) or with only one matching allele (bold, predicted semi-compatible).

Predicted Incompatible				Predicted Semi-compatible	
Family 8-1		Family 25-4			
Genotype	Fruit Set	Genotype	Fruit Set	Genotypes	Fruit Set
*S*8*a S*1*c*×*S*8*a S*1*c*	0/7	*S*25*aS*4*a*×*S*25*aS*4*a*	0/32	*S*8*a * ***S*** **1** ***c***×*S*8*b * ***S*** **1** ***c***	7/7
*S*8*b S*1*d*×*S*8*b S*1*d*	0/5	*S*25*bS*4*a*×*S*25*bS*4*a*	8/16	*S*8*a * ***S*** **1d**×*S*8*b * ***S*** **1** ***d***	7/7
*S*8b *S*1*c*×*S*8*b S*1*c*	0/3	*S*25*aS*4*d*×*S*25*aS*4*d*	0/11	*S*4*a * ***S*** **25** ***a***×*S*4*d * ***S*** **25** ***a***	20/20
		*S*25*bS*4*d*×*S*25*bS*4*d*	12/18	***S*** **4a** *S*25*a*×***S*** **4** ***a*** * S*25*b*	12/12
				*S*4*a * ***S*** **25** ***b***×*S*4d ***S*** **25** ***b***	18/18
*total*	0/15		20/77		64/64

Parents of full sib Family 8-1 had genotypes *S8a S8b* and *S1c S1d*.

Parents of family 25-4 parental genotypes were *S25a S25b* and *S4a S4d*.

Predicted incompatible crosses failed to set fruit unless parents possessed allele *S25b* while all predicted semi-compatible crosses set fruit. Family names denote the plant numbers of the parents. For instance family 8-1 are offspring derived from crosses between plant 8 plant 1.

Autogamous fruit set was rare among greenhouse grown *A. munita* plants despite large deposits of self-pollen visible on stigmas. However, some autogamous seed set occurred on plant 25, the parent that carried the *S25b* allele, as well as in 3 of 21 of its progeny favoring the hypothesis that *S25b* is a weak incompatibility allele. Occasional fruit set was also observed among un-genotyped bagged and hand self-pollinated individuals of *A. munita* (7 of 61 plants) indicating either additional variation in the strength of SI or that allele *S25b* was present among these plants. Outcross hand pollination from non-sib donors nearly always set fruit (136 of 141 pollinations). None of the F2 offspring from crosses among siblings germinated, perhaps due to inbreeding depression in addition to the already low rates of germination common in Papaveraceae. It was therefore impossible to test for expected segregation of parental sequences among F2 offspring.

### Hand pollinations – *Platystemon californicu*:

In contrast to *A. munita*, fruit set in *P. californicus* was never observed following hand or autogamous self-pollination but occasional fruit set did occur after putatively incompatible crosses. Prior to genotyping the progeny from two full sib families (1–2 and 1–3), we identified two stigma-S sequences from each parent: *S1a* and *S1b* from parent 1, *S2d* and *S2e* from parent 2, and *S3a* and *S3b* from parent 3. However, in both crosses, we later found that some of the putative *S*-sequences failed to segregate independently, but rather co-segregated as if linked. In family 1-3, sequences *S3a* and *S3b* (hereafter *S3a,b* where *a* and *b* are unique PCR products) were both found in 12 of 22 individuals and never found separately, ruling out that they are in fact separate alleles of a heterozygous *S*-locus as had been assumed when the cross was performed. A third product, *S3c*, was found but only observed in the absence of *S3a,b*. No homologous product linked to *S3c* was detected. Sequences *S1a* and *S1b* from the other parent in this cross were never found together in F1 progeny, characteristic of alleles segregating at a heterozygous *S*-locus. Family 1-2 also possessed a similar co-segregating polymorphism among F1 progeny. The PCR products *S2d and S2e* (hereafter *S2d,e*) from the male parent of this family were found together in 13 of 22 individuals (Table S1), while no sequence from plant 2 was found in the remaining 9 offspring. We apparently failed to amplify the alternative allele in this case.

Hand pollinations within the two families resulted in 93% fruit set when parents had one non-matching allele, while only 12% of crosses among plants with matching putative *S*-locus genotypes set fruit (Table 2). In *P. californicus*, a total of 16 fruits were set after putatively incompatible crosses. Ten of these fruits occurred in crosses among sibs from Family 1-3 where only one parental allele (*S1b*) was amplified and no sequence contributed by parent 3 was recovered (denoted S1b*×S1b* in Table 2). Fruit set among these offspring may have been due to failure to correctly genotype some individuals. If crosses among these ambiguous genotypes are excluded, only 6 of 108 pollinations among putatively incompatible individuals set fruit.

**Table 2:** Fruit set following hand pollinations among full sibs of *Platystemon californicus* with matching genotypes (predicted incompatible crosses) or one non-matching allele (bold, predicted semi-compatible crosses).

Predicted Incompatible				Predicted Semi-compatible	
Family 1-3		Family 1-2			
Genotype	Fruit Set	Genotype	Fruit Set	Genotypes	Fruit Set
*S*1*a S*3*a,b×S*1*a S*3*a,b*	0/33	*S*1*a S*2*d,e×S*1*a S*2*d,e*	4/18	***S*** **1** ***a*** * S*3*a,b*×***S*** **1** ***a*** * S*3*c*	16/17
*S*1*b S*3*a,b×S*1*b S*3*a,b*	0/17	*S*1*b S*2*d,e×S*1*b S*2*d,e*	1/13	***S*** **1** ***b*** * S*3*a,b*×***S*** **1** ***b*** * S*3*c*	18/20
*S*1*a S*3*c×S*1*a S*3*c*	1/8	(F2) *S*1*a S*1*b×S*1*a S*1*b*	0/17	*S*1*a * ***S*** **3** ***a,b***×*S*1*b * ***S*** **3** ***a,b***	7/7
*S*1*b* * *×S*1*b* *	10/23			*S* **1** ***b*** * S*2*d,e*×*S* **1** ***b*** * S*2?	5/5
				*S*1*a * ***S*** **2** ***d,e***×*S*1*b * ***S*** **2** ***d,e***	10/11
*total*	11/81		5/48		56/60

Parent 1 was genotyped *S*1*a* S1*b*, parent 3 was *S*3*a,b S*3*c* where *S*3*a,b* appears to be a linked polymorphism and *S*3*c* is allelic to one or both *S*3*a,b* sequences (see text).

Parent 2 was genotyped *S*2*d,e S*2*?* where sequences *d,e* were always either inherited together in F1's or, when neither was present, no alternative product was found.

*indicates individuals where only one *S*-like sequence was recorded.

F2 genotype *S*1*a S*1*b* was derived from predicted semi-compatible crosses among full-sibs from family 1-2.

Family names as in Table 1.

Fruit set following 22 hand self-pollinations in *P. californicus* was never observed (data not shown) nor was there any autogamous fruit set of bagged flowers despite copious autogamous self-pollen on stigmas. Germination of seeds from one cross among F1's with non-matching genotypes was sufficient to perform crosses among F2's. In Family 1-2, F2 crosses among offspring with the *S1a S1b* heterozygous genotype produced no fruit (N = 17, Table 2). In both *A. munita* and *P. californicus*, fruit set was significantly higher when non-matching rather than matching genotypes were crossed (Fisher's exact tests: *A. munita*, χ^2^ = 93.02 (1 *df*), *p*<0.001; *P. californicus* χ^2^ = 113.74 (1 *df*) *p*<0.001).

The finding of more than two *S*-like sequences from single individuals presents the possibility of a multi-locus system in which more than four incompatibility phenotypes might result from a cross. However, we always found incompatible pairs of plants in diallels among five full sibs indicating no more than four incompatibility phenotypes were present in each group of five sibs. Three full-sib 5×5 crossing arrays of *A. munita* all had at least one pair of individuals that were reciprocally incompatible (data not shown). An additional array possessed one pair of plants where three pollinations in one direction failed to produce fruit but the pair was not crossed reciprocally. In *P. californicus*, four arrays among full-sib family 1-3 and two among family 1-2 each possessed at least one pair of reciprocally incompatible plants. While this test cannot rule out that more than four mating types are present in a full sibship, we found no evidence of more than four mating types in any of the ten sets of five sibs tested, consistent with expectations of single-locus GSI.

### Tissue specific RT-PCR

**Figure 2:**
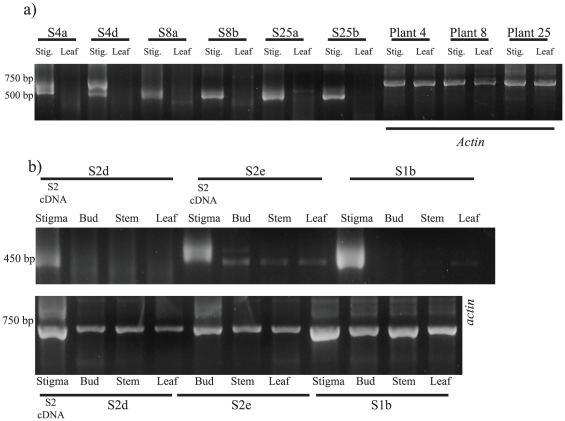
RT-PCR of putative *S*-locus genes from stigmatic, stem and leaf tissues using allele-specific forward primers and a universal amplification reverse primer. (a) Expression of *A. munita* sequences was detected in stigmatic but not leaf tissue. (b) Expression in *P. californicus* tissues is highest in stigmas as expected for *S*-alleles. *Actin* primers were used as positive controls for all plant tissue cDNA from both species. Putative alleles *S2d* and *S2e* appear to be linked. Sequencing of bands in non-stigmatic tissue using *S2e* primers showed that they are products not previously identified from stigmas. *Actin* primers were used only once on plant S2 stigmatic tissue, hence there is one less lane in the *actin* gel than in the allele specific gel.

We investigated the expression of putative *S*-alleles in various tissues by allele specific amplification of cDNA (Figure 2). Overall, the expression of putative *S*-alleles was mostly localized to the stigma. The *A. munita*, sequences *S*4*a*, *S*4*d*, *S*8*a*, *S*8*b*, *S*25*a* and *S25d* showed no expression in leaves but appear to be highly expressed in stigmatic tissue (Figure 2). Other tissue types were not tested for this species. In *P. californicus*, we used an individual (genotype *S1b S2d,e*) from the crossing family 1-2 (Table 2). Alleles *S1b* and *S2d* showed high expression in the stigma as expected for a *S*-allele, with a faint band appearing in the leaf tissue for allele *S1b* (Figure 2*b*). Allele *S*2*e*, a potential paralog linked to *S2d*, appeared to show weak expression in unopened buds, stems, and leaves, as well as in the stigma. However, sequencing of this amplicon from non-stigmatic tissue types revealed that it was not *S2e* but a potential paralog not previously identified from the parental stigmas.

### Phylogeny

**Figure 3:**
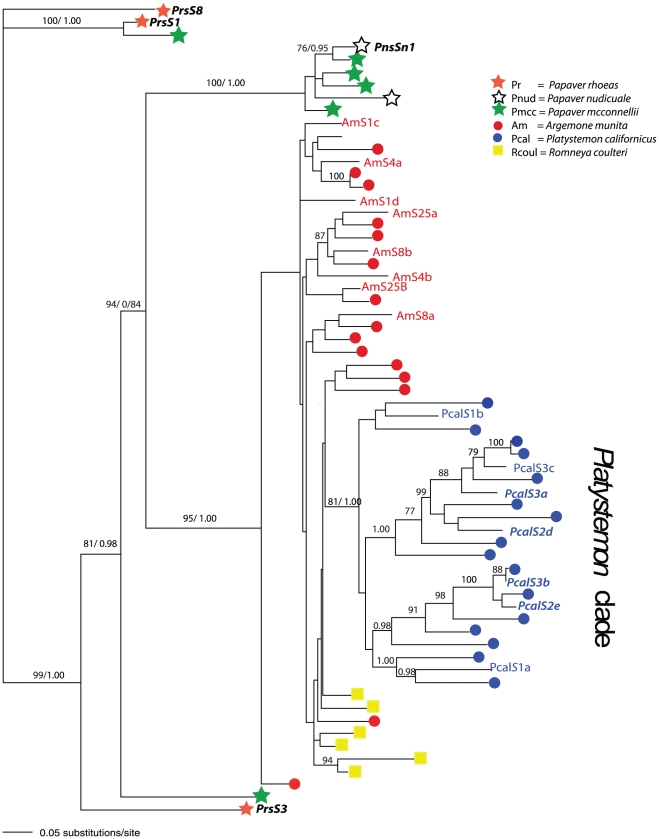
Maximum likelihood phylogeny of known *Papaver S*-alleles and putative *S*-alleles from other taxa. The *Papaver S*-alleles (alleles *PrsS1*, *PrsS3 PrsS8* and *PnSn1* in bold) identified by [5] and [6] form highly divergent lineages that include recently identified sequences from *P. mcconnellii* but show no close associations with any non-*Papaver* sequences. All sequences from *P. californicus* form a distinct monophyletic clade while sequences from *A. munita* and *R. coulteri* show some poorly supported shared polymorphism. *P. californicus* linked sequences *S2d* and *S2e* from parental plant 2 are found in separate sub-clades as are co-inherited sequences *S3a* and *S3b* from parent 3 (italics). Sequences used in crosses for *A. munita* and *P. californicus* are indicated as text.

Phylogenetic reconstruction of sequences from *Papaver*, *Argemone*, *Platystemon* and *Romneya* shows interesting features (Figure 3). The previously isolated *S*-alleles from *Papaver* exhibit no shared ancestral polymorphism with non-*Papaver* sequences. The newly identified *P. mcconnellii* and *P. nudicaule* alleles show some trans-specific polymorphism with both *P. rhoeas* and *P. nudicuale*, but nevertheless cluster together with congeneric sequences rather than with sequences derived from other genera.

Sequences from *P. californicus* form a distinct, well-supported clade and show no evidence of shared polymorphism with other taxa (Figure 3). Linked sequences *S2d* and *S2e* from the two crossing families are found in separate sub-clades within the *P. californicus* lineage as are the linked homologs *S3a* and S3b. *A. munita* and *R. coulteri* sequences show some possible shared ancestral polymorphism but these lineages are not well resolved. Overall, there is little phylogenetic structure among sequences from *A. munita* compared to those from *P. californicus*. A phylogeny including *Arabidopsis thaliana S*-protein homologs (SPHs) shows that the *A. thaliana* SPH sequences form a distinct clade relative to all Papaveraceae sequences (Figure S1). This suggests that our putative *S*-alleles are most closely related to functional *P. rhoeas S*-alleles and unlikely to be orthologous to any other known members of the SPH gene-family.

### Sequence variability

**Figure 4:**
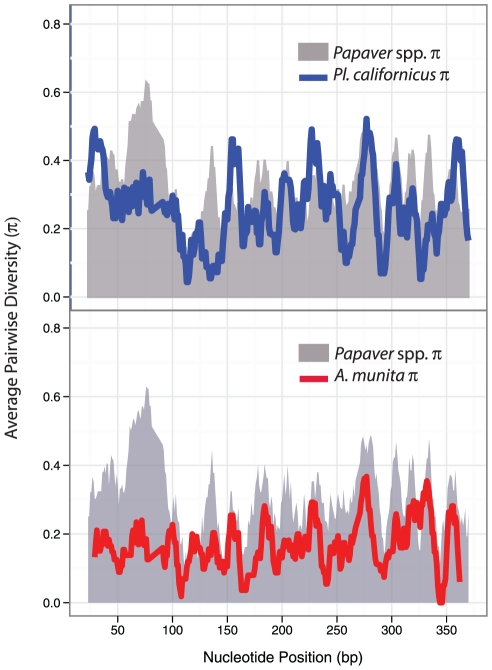
Sliding window analysis of average pairwise nucleotide diversity (π) of putative *S*-alleles. Data consist of 10 *Papaver* spp (gray), 11 *P. californicus* (blue), and 11 *A. munita* (red) sequences possessing 5′ ends. Regions of high divergence across the three genera appear in similar positions. Divergence was estimated using DNASP [72].

Average pairwise estimates of synonymous, non-synonymous and total nucleotide divergence (π) show that the previously sequenced *S*-alleles from *Papaver* are the most variable (Table 3). Average divergence in *Papaver* is reduced slightly when sequences from *P. mcconnellii* are included due to the clustering of several sequences from this species with the previously published allele from *P. nudicaule* (see Figure 3). Sliding window analysis of 5′ sequence alignments show variation at similar positions across taxa but the amount of variation differs, with a region between 50–100 bp in *Papaver* showing the greatest diversity (Figure 4). Average pairwise diversity for the *P. californicus* sample is greater overall than for *A. munita*.

**Table 3:** Average pairwise nucleotide divergence among sequences within genera.

	Synonymous (Π_s_)	Non-Synonymous (Π_a_)	All Sites (Π)
*Papaver* (S1, S3, S8, Sn1)	0.70	0.28	0.36
*Papaver* (all)	0.57	0.24	0.30
*A. munita*	0.30	0.14	0.17
*P. californicus*	0.49	0.20	0.26
*R. coulteri*	0.20	0.11	0.13

Only sequences with 5′ ends were included except *R. coulteri* where only 3′ end sequences were amplified. The four known *Papaver S*-alleles have the greatest overall divergence for both types of sites.

### Estimates of selection

Putative *S*-allele sequences appear to be under positive selection. Likelihood ratio tests show that models with positive selection fit the observed data better than neutral models (Table 4). This holds for the genera with lower sequence diversity even after *Papaver* spp. sequences are removed. The empirical Bayesian method [33] identified only a few sites with statistically significant evidence for positive selection, while the majority of sites are found to be under purifying selection (Table 4). Using sequences with complete 5′ ends, datasets including and excluding *Papaver* sequences were analyzed to determine their effects on estimates of selection in *A. munita* and *P. californicus*. Neutral null models M1 and M7 that do not allow for positive selection were rejected using likelihood ratio tests against the alternative models M2 (two rate model), M3 (three discrete rates model), and M8 (beta distribution+ω model). Posterior probability scores indicate sites 53, 107 and 119 are under selection under models M2 and M8. The discrete M3 model shows no sites under positive selection when *Papaver* is present but that 6 sites (sites 53, 77, 83, 103, 107, and 119) have high posterior scores when those sequences are excluded. Model M8 shows higher mean dN/dS when *Papaver* is excluded suggesting those highly divergent sequences may have a dampening effect on estimating sites under selection.

**Table 4:** Likelihood ratio test statistics (−2ΔlnL) compare models with selection (boldface) to preceding neutral models on 5′ end containing sequences from *Papaver*, *Argemone* and *Platystemon*.

a)				
Model	Likelihood	−2ΔlnL	*p*-value (df)	Selected sites
M1	−6410.82			N/A
**M2**	−6406.15	9.34	*p*<0.01 (2)	107, 119
**M3**	−6374.01	73.62	*p*<<0.01 (3)	None
M7	−6370.63			N/A
**M8**	−6366.94	7.38	*p*<0.05 (2)	107*, 119

b)				
Model	Likelihood	−2ΔlnL	*p*-value (df)	Selected sites
M1	−4055.71			N/A
**M2**	−4049.04	13.34	*p*<0.01 (2)	53, 107*, 119
**M3**	−4034.8	41.82	*p*<<0.01 (3)	53, 77, 83, 103, 107, 119
M7	−4037.95			N/A
**M8**	−4030.48	14.94	*p*<<0.01 (2)	53, 107*, 119

Posterior probabilities for sites under selection are ≥0.95 except when followed by an asterisk, in which case 0.90≤p≤0.95.

5′ Alignment of 10 *Papaver*, 11 *A. munita* and 11 *P.calfornicus* sequences (a). 5′Alignment of 11 *A. munita* and 11 *P.californicus* sequences only (b).

We also estimated selection using OmegaMap [34] because this method accounts for recombination (see Text S1 for analysis of recombination). Using sequences from *Papaver*, *Argemone* and *Platystemon*, this method finds more sites under selection (Figure 4). Codons 13, 33 and 68 were predicted to be under positive selection with OmegaMap but not PAML. Codons 107 and 119 were consistently predicted to be subject to positive selection using both methods, while codon 103 was predicted to be under positive selection in PAML model M3 and OmegaMap. Using OmegaMap, site 53 only reached high posterior probability (0.92) of positive selection after exclusion of *Papaver* sequences. It appears that the independent model of rate variation may lack power when only *A. munita* and *P. californicus* are included (22 sequences), as fewer sites are identified than when *Papaver* is included.

## Discussion

For all species examined, we amplified polymorphic sequences that resemble the four previously described *S*-alleles from *P. rhoeas* and *P. nudicaule* [5], [6]. Crossing experiments show that putative *S*-locus genotypes significantly predict fruit set in crosses among full-sibs of both *A. munita* and *P. californicus*, even in genotypes with co-segregating *S*-like sequences. In both species, crosses between parents with at least one non-matching allele nearly always set fruit, while crosses between individuals with matching putative *S*-locus genotypes displayed much lower rates of fruit set. In *A. munita*, fruit set following crosses among individuals with matching genotypes only occurred when allele *S25b* was present, suggesting that this may be a weak or non-functional *S*-allele. For *P. californicus*, although we cannot rule out leaky or partially expressed self-incompatibility [35]–[37], the majority of unexpected fruit set occurred in crosses among ambiguously genotyped individuals. In sum, sequences identified here and examined in crosses appear to either be functional *S*-alleles, or are linked to the functional genes, a distinction no crossing experiment can settle.

While two homologous products were found for several individuals of *A. munita* and *P. californicus*, as expected of an obligately heterozygous GSI locus, only one product was found for several individuals of these species and for all individuals of *Papaver*. The finding of only one *S*-like sequence in some plants may imply that amplification is inconsistent among genotypes, perhaps due to PCR competition among alleles, or that other lineages of *S*-alleles exist that are too divergent to be amplified using our degenerate forward or reverse primers. Despite crossing data to show that *P. rhoeas* possesses more than 60 *S*-alleles [4], Kurup et al. [6] were only able to sequence two alleles from *Papaver* using various hybridization and PCR techniques to add to the two previously described [5]. However, even in systems where the range of variation at the *S*-locus is well-characterized, amplification of fewer than two alleles from individuals is common in population studies [38], [39].

More than two sequences were found in some individuals of both *A. munita* (1 of 23 plants) and *P. californicus* (4 of 25 plants). In *A. munita* finding more than two *S*-like sequences in at least some individuals was not entirely surprising as this species is tetraploid. However, our crossing data so far find no indication of more than four mating types among full sibs suggesting one homologue of the *S*-locus may be deleted or non-functionalized in many individuals. The “extra” copy may have been amplified in the individual from which three sequences were recovered. While polyploidy, or even duplication of just the pollen-determining part of the *S*-locus causes loss of self-incompatibility in RNase-based SI of Solanaceae and Plantaginaceae, [40]–[42], this finding cannot be generalized to other GSI systems [43].

In diploid *P. californicus*, finding more than two copies of *S*-like genes was unexpected. Because *P. californicus* carries the chromosome complement considered basal in the family, it is unlikely to represent a recently diploidized polyploid. Rather, presence of multiple *S*-like sequences likely represents tandem duplication following divergence from *Papaver*. No linked or unlinked homologous *S*-like products have been detected in *P. rhoeas* (V.E. Franklin-Tong *personal communication*), despite considerable efforts to characterize its *S*-locus [44]. RT-PCR of RNA from *P. nudicaule* stigmas using primers designed to amplify sequences found in *A. munita* and *P. californicus* did not yield any sequences in *Papaver* closely similar to those from *A. munita*, *R. coulteri*, or *P. californicus* (data not shown). It is also unlikely that any of our sequences are orthologs of the *Arabidopsis* SPH proteins because phylogenetic estimates (Figure S1) indicate substantially closer homology to the demonstrated *Papaver S*-alleles.

Duplication of *S*-locus genes is apparently common in SI systems. In the sporophytic system of the Brassicaceae, homologous polymorphic stigmatic *S*-locus genes *SRK* (*S-receptor kinase*) and *SLG* (*S*-locus glycoprotein) are found in *Brassica oleracea*, *B. campestris* and *Raphanus sativas* [16], [45], [46] while only *SRK* is found in *Arabidopsis lyrata*, *A. halleri* and *Capsella grandiflora* [22], [25], [26], [47]–[49]. *SLG* was either lost in some ancestor of both *Arabidopsis* and *Capsella* or gained in *Brassica/Raphanus*. Recently, Busch et al. [50] reported several loci in *Leavenworthia alabamica* that are similar to *SRK*, only one of which is shown to co-segregate with SI. Duplications are also abundant in the *S*-RNase system where multiple pollen-*S*-like F-Box genes are found linked to the *S*-locus in some taxa [51]–[53]. The presence of multiple linked loci makes it difficult to isolate the genes directly involved in SI [54]–[55], and recent evidence [56] strongly suggests that, at least in some cases, linked loci may act jointly to determine specificity.

The expectation for loci under balancing selection is that polymorphism will be preserved over very long time periods resulting in trans-specific polymorphism. This seems to be true of the sequences from *Papaver* which display several diverse and apparently old lineages. However, for the other taxa sampled, our phylogenetic analysis indicates a general clustering of putative *S*-alleles according to genera, relatively little phylogenetic depth to the sequences from each genus, and the lack of trans-generic lineages with the only minor exceptions being among poorly supported nodes subtending sequences from *A. munita* and *R. coulteri*.

There several potential reasons why shared ancestral polymorphism is largely absent from the sequences found here. First, the primers used in our search for S-alleles were based on the four published allele sequences from *Papaver*. Additional sequence diversity may exist within *Papaver* which could explain why studies prior to ours failed to amplify additional *Papaver* S-alleles from *P. rheoas*. It could be that S-locus diversity outside that known from *Papaver* exists in the taxa studied here and its discovery could alter our view of the extent and distribution of ancient polymorphism. Nevertheless, the level of polymorphism in sequences amplifiable with existing primers appears considerably smaller outside of the genus *Papaver* than within it.

Second, the sequences outside of *Papaver* might not represent functional *S*-alleles, but rather paralogs of the *S*-locus not directly involved in incompatibility. The strength of balancing selection acting on such paralogs, even if linked to the *S*-locus as found here, would be much reduced relative to functional *S*-alleles, perhaps explaining their reduced levels of divergence, shorter coalescence times, and lack of shared ancestral polymorphism.

Third, lack of shared ancestral polymorphism may reflect long divergence times among the genera examined (Table S2), with corresponding turnover among the alleles maintained in each genus. Castric and Vekemans [48] highlighted a nearly monophyletic clade of *Brassica oleracea SRK* alleles with only two trans-specific lineages shared with *Arabidopsis lyrata* and none with *A. halleri*. This was explained by the 15–20 million year divergence of *B. oleracea* from *Arabidopsis* [57] resulting in lineage sorting. Among families with RNase-based GSI, contrasting patterns are seen. In the Solanaceae, despite an estimated 40 million years of divergence among sampled genera [23], ancestral polymorphism is widely shared. In the Rosaceae, reciprocally monophyletic sets of alleles are observed from subfamilies Prunoideae and Maloideae [58] thought to have diverged some 32 MYA [59]. Minimum divergence time estimates among the genera examined here (10–40 MY; Table S2) appear at least as long as those between SSI *Arabidopsis* and *Brassica*. Following lineage sorting some variation in observed coalescence times of sequences from different species is expected, though the variation seen here between, for example, *Argemone* and *Papaver* appears extreme.

Fourth, reduced long-term population sizes and/or population bottlenecks can increase the rate of turnover affecting both levels of shared ancestral polymorphism and sequence variation found in loci under diversifying selection. For *S*-alleles, two cases of ancestral restrictions of *S*-locus diversity have been described [21], [23], [24]. However, the single monophyletic clade of alleles from *P. californicus*, and the nearly monophyletic lineage from *A. munita* are unlikely to be attributable to past bottlenecks. At a gametophytic *S*-locus, a self-incompatible population cannot persist with fewer than three alleles [60] so bottlenecks resulting in a single surviving lineage would be unexpected. In addition, one would have to invoke separate bottlenecks, or a bottleneck followed by turnover, to explain the reciprocally monophyletic sequences from *Platystemon* and *Argemone*/*Romneya*.

Finally, gene duplication and recombination/gene conversion could also cause the observed phylogenetic patterns. The expected consequence of recombination on genealogies of balanced polymorphisms is shorter times to coalescence and long terminal branches relative to depth, resulting in a topology with a star-like appearance and reduced phylogenetic structure [60]–[63]. Short coalescence time and lack of structure is particularly apparent in the sequences from *A. munita* suggesting that recombination might be particularly high in this taxon. While it is difficult to provide definitive tests for recombination when multiple homologous loci are present, preliminary analyses (Table S3, Figure S2) provide estimates of increased recombination in *A. munita* relative to *P. californicus*. A higher population recombination rate may explain the low phylogenetic resolution of sequences from *A. munita* relative to *P. californicus* while gene duplication and gene conversion in both of these taxa may explain both their low levels of variation and monophyletic or near-monophyletic clusters of sequences relative to *Papaver*. Using the *Brassica SRK-SLG* system as a model, Takuno *et al.* [64] demonstrated clear evidence for interlocus gene conversion. Simulations showed that when a functional SI gene is under diversifying selection, a duplicated copy can be advantageous if it contributes to variation in the functional gene through gene conversion.

**Figure 5:**
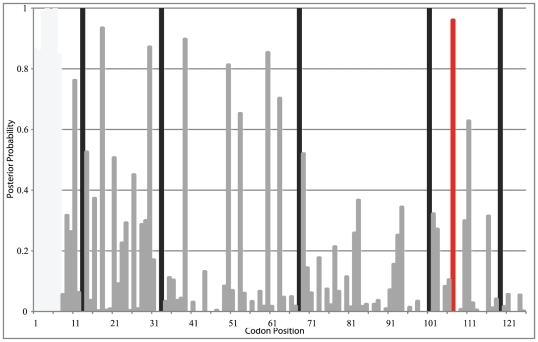
Posterior probabilities for codons estmatied to be under positive selection using OmegaMap [**34**] implemented with an independent model of rate variation for each site. The dataset used includes 10 *Papaver*, 11 *P. californicus* and 11 *A. munita* sequences. Sites 1–7 from *A. munita* and *P. californicus* are excluded because they are not present in *Papaver*. Codons 13, 33, 68, 103, 119 (black bars) and 107 (red) are predicted to be under positive selection.

Preliminary codon based estimates of selection (Table 4, Figure 5) suggest substantial constraint with several residues under positive selection. The secondary structures of the *Papaver* stigmatic *S*-proteins *PrsS1*, *PrsS3*, *PrsS8* and *PnudSn1* [14] predicted seven hydrophilic surface loops. Substitution at a single variable site within loop 6 of *PrsS1* caused loss of function. This amino acid corresponds to site 107 in our alignment (Figure 1) and attains high posterior probability of positive selection in all analyses. This functional assay counters the criticism that the types of statistical methods used here are unreliable in detecting important functional regions undergoing diversifying selection [65]–[67].

We have presented putative stigmatic *S*-locus sequences from several naturally occurring species of Papaveraceae. It is not yet proven that any of these sequences function directly in self-pollen recognition and rejection. However, their homology to the known *S*-alleles of *P. rhoeas* and the correspondence between genotype and incompatibility phenotype in our test crosses suggest that many may function as S-alleles or, at the very least, are linked to the *S*-locus in these taxa. Future studies that use *in vitro* assays of the effects of expressed proteins on pollen tube inhibition [5], [6] are needed to unambiguously determine function and identify potentially non-functional homologs. Preliminary sequence analyses also show evidence of positive selection, as expected for S-alleles. The phylogenetic relationships, with much lower levels of sequence diversity displayed by *A. munita* and *P. californicus* relative to the levels of polymorphism among confirmed *S*-alleles from *Papaver* are somewhat surprising, though not unprecedented among loci under balancing selection. If observed levels of polymorphism represent real differences at the *S*-locus among the genera reported here, then molecular details of the *S*-locus, and the genome in which it resides, for instance whether or not there are duplicate copies of *S*-locus genes, may play a role in shaping patterns of polymorphism at loci under balancing selection.

## Materials and Methods

### Plant Material

Stigmatic tissue of *P. californicus* was collected from randomly sampled individuals from three previously documented populations ([30] and G. Hannan *personal communication*): Lake Cuyamaca State Park east of San Diego (N 32°58′59.1″, W 116°33′45.4″) Hastings Natural History Reservation (N 36°23′07.1″, W 121°33′16.6″; UC Reserve Permit: 5870), and Carmel Valley Road near Carmel, CA (N 36°26′34.5″, W 121°38′53.7″). Seeds of *P. californicus* were also collected from the Hastings Natural History Reservation and germinated for greenhouse crossing experiments. *Argemone munita* stigmatic tissue and seeds were collected near Valentine Eastern Sierra Reserve, Mammoth Lakes (N 37°36′51″, W 118°49′47″; UC Reserve Permit: 6370), CA and Emerson Oaks UC Reserve near Temecula, CA (N 33.466668, W -117.03944; UC Reserve Permit: 6723). *Romneya coulteri* stigmas and leaves were collected from plants growing in Del Mar, CA, Otay Mesa near San Diego, and on the UCSD campus. *P. nudicaule* (Iceland Poppy) was obtained from a commercial seed packet. *P. mcconnellii* was collected from a steep rocky scree outcrop near Highway Pass in Central Alaska (approx. N 63°28′, W 150°10′).

Bulk seed of *A. munita* collected from wild populations from San Diego County was used for greenhouse crosses. Seeds were sown individually in 1″ plug trays in moist SunGro Professional mix. Germination rates were very low for both *A. munita* and *P. californicus*, which typically required a minimum of 45–90 day cold treatment (4°C) before any seedlings emerged. *A. munita* was grown from seedlings in a glasshouse with natural light until flowering when hand pollinations were conducted. After germination, *P. californicus* was grown to flowering under fluorescent lights at 14/10 hr day/night light regimes. Full-sib families of both *A. munita* and *P. californicus* were produced from genotyped parental plants. Seeds of *P. mcconnellii* were cold stratified (4°C with 6 hr light) for one month to induce germination, and plants were grown in 1∶1∶1 perlite∶vermiculite∶coconut coir in a glasshouse with natural light.

For *A. munita* and *P. californicus*, hand pollinations among sets of full-sib offspring were used to test the relationship between putative *S*-locus genotype and incompatibility phenotype. Flowers of both species were bagged prior to anthesis to prevent pollen contamination. Replicate pollinations of three or more crosses between each reciprocal pair were attempted, though not always feasible due to limited flowers. Pollinations were made by collecting anthers using a forceps, and depositing pollen on the stigma. Crosses were made among individuals with matching sets of sequences (‘incompatible’ treatment) and those that differed at a single putative allele (‘semi-compatible’ treatment). Self and non-matching genotype crosses were conducted in similar fashion.

The finding of more than two *S*-like sequences from single plants (see results) presented the possibility of a multi-locus system of self-recognition. To test for this we conducted ten 5×5 reciprocal diallel crosses (four in *A. munita* and six in *P. californicus*) among full-sib offspring. In a single-locus system, only four mating types are expected among full sibs so diallel crosses among five full siblings should always uncover at least one incompatible pair. In multi-locus systems, the number of mating types among full sibs is expected to be greater, 16 if there are two heterozygous loci which both act to confer phenotype and so diallels among five sibs might often uncover no incompatible pairs. These diallel crosses were conducted in the absence of genotypic data for the individuals used.

### Molecular Techniques

Amplification of putative *S*-alleles was performed using reverse-transcriptase (RT-) PCR from total RNA extracted from stylar/stigmatic tissue. RNA was extracted using Trizol (Invitrogen Corp.) or the PureScript kit (Gentra Systems Inc., Minneapolis, MN). For *Papaver* spp. some sequences were amplified from genomic DNA extracted from leaf tissues with the CTAB method [68] or with PUREGENE DNA purification kit (Gentra Systems Inc., Minneapolis, MN).

The forward primers Prho1F: 5′ATG/AAY/MRR/MGR/GGN/AAY/GG and Prho4F: GTG/CGH/ATA/ATG/AAY/ARR/AGA/GG were designed from a conserved amino acid region based on the four published *Papaver* sequences and annealed to a region approximately 90 bp downstream from 5′ end. These were used in conjunction with an oligo-dT reverse primer (AUAP, Invitrogen Corp.) for amplification of 3′ ends of putative *S*-alleles from cDNA. Nested PCR was performed for several individuals of *P. californicus* due to non-specific amplification using the forward and reverse degenerate primers Prho1F and PopR3: GCC/ASR/TRH/ADV/ASC/WGC/C. Amplified PCR products were then sub-cloned into competent *E. coli* cells using the TOPO 2.1 TA cloning kit (Invitrogen Corp.). Restriction fragment analysis was used to identify unique products from clones.

Because there is an approximately 90 bp region upstream from the forward degenerate primers in the 4 published *Papaver* sequences, 5′ rapid amplification of cDNA ends (5′ RACE) was performed using the Roche 2^nd^ Generation 5′ RACE kit (Roche Diagnostics). RNA was first transcribed into cDNA using allele-specific reverse primers obtained from 3′ end sequence data. This technique used the enzyme terminal transferase to ligate a poly-A region to the 5′ end of cDNA and then one or two nested PCR reactions using additional allele-specific reverse primers. To obtain full-length *Papaver spp.* sequences, genomic DNA was fragmented with a restriction digest, and an adapter was attached to the ends. PCR was conducted with an allele specific primer and a generic primer on the adapter.

To assess tissue specificity of expression of putative *S*-alleles, RNA was isolated from stigmas, leaves, stems and buds of unopened flowers. Total RNA was treated with Ambion Turbo DNase (Ambion Inc.) according to the supplier's protocol to remove genomic DNA. Extractions were tested for DNA contamination by performing allele specific PCR prior to reverse-transcribing to cDNA. Because it is not possible to completely remove all genomic DNA, we used allele specific forward primers and a reverse abridged universal amplification primer that anneals downstream of the poly-A tail on samples suspected to have remaining DNA. The quality of cDNA was then tested by PCR using *actin* primers.

### Sequence Analysis and Phylogenetics

Sequences were aligned with BioEdit v7.05 (Ibis Therapeutics, Inc.) using the known *P. rhoeas* stigmatic *S*-alleles as a guide. A total of 64 sequences were used to construct the phylogeny including sequences with 5′ and 3′ ends and those with only 3′ ends. A maximum likelihood (ML) phylogenetic tree was constructed using PAUP [69] under the GTR+I+G substitution model. ModelTest 3.7 [70] was used to determine base frequencies (A = 0.314 C = 0.153 G = 0.244 T = 0.287) and the appropriate substitution model. Branch support for the ML tree was obtained by bootstrap analysis of 1000 replicates using the same base frequencies as above. Mr. BAYES [71] was also used to confirm the maximum likelihood topology and branch support. The Bayesian analysis was run using a GTR+I+G substitution model across sites for 1,000,000 generations, sampling every 100^th^ tree for a total of 10,000 trees. A burn-in of 2500 trees was discarded and posterior probabilities were calculated on the remaining 7500 trees. No root was specified for either ML or Bayesian analysis but the *P. rhoeas* S1 and S8 alleles were found to form distant sister group to all other alleles. Since the ML and Bayesian methods produced similar topologies, only the ML tree is reported.

### Sequence diversity

Average pairwise nucleotide divergence was estimated using DNASP [72]. Sliding window estimates of interspecific divergence were estimated for alignments of full-length sequences (including the 5′ region). The 5′ dataset included 10 full-length *Papaver*, 11 *P. californicus* and 11 *A. munita* sequences including 5′ ends, but with incomplete data for approximately 30 nucleotides at the 3′ end.

### Estimates of positive selection

To estimate the ratio (ω) of non-synonymous (d_N_) to synonymous (d_S_) substitution rates at each amino acid position we used the program *codeml* in PAML 3.15 [72]. Values of ω<1 for individual codons indicate purifying selection while sites with ω = 1 are considered neutral. Positive selection at the amino acid level is inferred when ω>1. Likelihood ratio tests are used to compare a series of nested neutral and selection models [32], [73] to determine the model that best fits the data. The null model M1 (neutral) constrains all sites to be either of class ω = 0 or ω = 1 while the alternative model M2 adds a third class in which ω is free to vary at individual sites. Model M3 assumes three discrete site classes (ω_0_<1, ω_1_ = 1 and ω_2_>1) with three corresponding proportions (*p*_0_, *p*_1_, *p*_2_) estimated from the data. Two similar models, M7 (neutral) and the alternative M8 (selection) assume a beta distribution of rates among 11 site classes. Sites estimated to be under positive selection are determined by an empirical Bayesian approach [73] where posterior probabilities are estimated from rates within each site class. Because individual species possess several polymorphic alleles, comparisons of d_N_/d_S_ ratios can be made within and between species and can also be estimated cumulatively [32], [74]–[76].

We also used OmegaMap [33] to determine whether estimates of selection on individual codons differ using a coalescent method that allows for recombination. An independent model for ω at each site was used for 5′ datasets, as these were sufficiently small (32 and 22 sequences, with and without *Papaver* spp., respectively, to allow for this computationally intensive MCMC chain of 500,000 generations. An improper inverse prior distribution of ω was set for the independent model.

## Supporting Information

Figure S1 **Bayesian phylogeny of** ***Papaver*** **S-alleles (** ***PrS1*** **,** ***PrS3*** **,** ***PrS8*** **, and** ***PnSn1*** **) and 28 putative Papaveraceae** ***S*** **-alleles showing their relationship to 6** ***Arabidopsis thaliana*** **S-protein homologues (SPH's).** The SPH's were chosen using a maximum E-value of 10^−4^ in tBLASTx searches against the *A. thaliana* protein database. Numbers on nodes are posterior probability scores. The phylogeny is midpoint rooted but this did not effect distances between sequences. *Argemone munita* sequence S25a queried against the Flowering Plants database resulted in E-values ranging from 9e^−16^ to 5e^−20^ with closest hits to the four *Papaver* S-alleles. Similar results are obtained using any other sequence from our sample. SPH sequences were obtained from NCBI accessions 18390419, 18414536, 18416755, 22328708, 21404932 and 22328912.
(DOC)

Figure S2 **Coalescent simulation of the effect of sequences from a paralogous gene on recombination detection.** Some putative S-alleles in our data set may belong to one or more duplicated, paralogous S-pseudogene. These are not expected to be under balancing selection and are likely to be released from recombination suppression. Therefore, even if functional *S*-alleles experience complete recombination suppression, contamination of the dataset by recombining *S*-pseudogenes could cause statistically detectable recombination. The goal of this simulation was to evaluate the effect of *S*-pseudogene haplotypes on the detection of recombination in a combined data set. We conducted coalescent simulations with Hudson's [77] ms program, modified to allow changes in recombination rate over time. The allelic genealogy of S-alleles can be approximated with a neutral coalescent with a scaling factor, *f* [78]. We assumed that the population parameter, *θ_s_*, of *S*-alleles to be *4 f N_e_ μ*, where *N_e_* is the neutral effective population size and *μ* is a neutral mutation rate. For the paralogous pseudo-gene, which has been released from balancing selection, the population parameter was set to that of a neutral gene (*θ_p_ = 4 N_e_ μ*). At *t_d_* generations ago (in units of *4 N_e_ f*), there was a duplication of an S-allele and a paralogous pseudo-gene was created. We assumed that functional S-alleles do not recombine (the gray area), but the pseudo gene is released from recombination suppression and recombination within the paralogous alleles can occur immediately after the gene duplication (black region) with the recombination rate between the neighboring nucleotide sites of *r* per generation. Since we assumed that the pseudo gene was created by a single duplication event, we forced the S-pseudogene to have a severe bottleneck of *θ_b_* for *t_b_* generations (in units of *4 N_e_ f*). In the coalescent simulation, we assumed that we have sampled 24 sequences, of which some proportion, *c* (25%, 50%, or 75%), is actually contamination from the S-pseudogene. For each genealogical tree created by the coalescent simulation, DNA sequence evolution was simulated by Seq-Gen version 1.3.2 [79] under the Jukes-Cantor model [80], the non-parametric permutation tests of recombination were conducted with permute (r^2^ statistics) in OmegaMap v0.5 [33] and we recorded the proportion of simulations with the *p*-value≤0.05. We assumed a sequence length of 327 bp, which is close to that of our data, and the following parameters were used: *f* = 20; *θ_s_* per site = 0.01, 0.1, 0.2, 0.3, 1, or 2; *t_d_* = 0.01, 0.1, 0.1, 1, 2, or 3; *r/μ* = 2, 20, 60, 100, 200, or 2000; *t_b_* = 0.005; *θ_b_* per site = *θ_s_*/2000000. All factorial combinations of the parameter values were used, and 1000 simulations were run for each set. In our simulations, the probability of detecting recombination with the nonparametric permutation did not exceed 0.075. This is because compared to the large diversity of S-alleles, the newly duplicated pseudo-gene has a low diversity and recombination within the pseudogene does not contribute much to the detection of recombination. These results suggest that it is unlikely that the recombination detected in our dataset is due to inclusion of S-pseudogenes.
(DOC)

Table S1 **Segregation of parental alleles among full sibs used for testing the relationship between putative SI genotype and phenotype.** Tests compare expectations for two alleles from each parent segregating separately to create four offspring genotypes. For crosses involving *Pl. californicus*, two sequences from one parent were always found together in offspring or else neither was found. These are considered linked polymorphisms (21a,b) and (26a,b). From plant 26, an alternative allele (26c) was recovered in offspring where 26a,b was absent (see main text).
(DOC)

Table S2 **Estimated divergence times among taxa.** Divergence times were estimated as the number of silent substitutions per site divided by the silent substitution rate (substitutions per site per year). Three genes were used for these estimates: the ribosomal internal transcribed spacer (ITS) and chloroplast genes (*rbc*L and *atp*B). Sequences were obtained from NCBI (AF057672, AF098922, AF305331, DQ912885, U86621, U86632, U86630, FJ626614, U86393, U86394, U86395, U86399), and the number of silent substitutions was estimated from DNASP [69]. Several estimates of angiosperm silent substitution rates were obtained for each gene [81]–[83], and the most extreme values were used for the min and max estimates of Papaveraceae divergence times.
(DOC)

Table S3 **Non-parametric permutation analyses of the correlation between linkage disequilibrium and distance (top) and coalescent likelihood estimates of the population mutation (θ = 4N_e_μ) and recombination (ρ = 4N_e_r) rates with 95% highest posterior densities (bottom).**
(DOC)

Text S1 **Methods describing estimation of recombination rates in** ***A. munita and P. californicus*** **.**
(DOC)
