# Supplementary material for: Evolutionary Genetics of an S-Like Polymorphism in Papaveraceae with Putative Function in Self-Incompatibility
Source: PLoS One. 2011 Aug 31;6(8):e23635. doi: 10.1371/journal.pone.0023635 (PMC3166141; doi:10.1371/journal.pone.0023635)
Supplement: Figure S1 — Bayesian phylogeny of Papaver S-alleles ( PrS1 , PrS3 , PrS8 , and PnSn1 ) and 28 putative Papaveraceae S -alleles showing their relationship to 6 Arabidopsis thaliana S-protein homologues (SPH's). The SPH's were chosen using a maximum E-value of 10−4 in tBLASTx searches against the A. thaliana protein database. Numbers on nodes are posterior probability scores. The phylogeny is midpoint rooted but this did not effect distances between sequences. Argemone munita sequence S25a queried against the Flowering Plants database resulted in E-values ranging from 9e−16 to 5e−20 with closest hits to the four Papaver S-alleles. Similar results are obtained using any other sequence from our sample. SPH sequences were obtained from NCBI accessions 18390419, 18414536, 18416755, 22328708, 21404932 and 22328912. (DOC) [file pone.0023635.s001.doc]

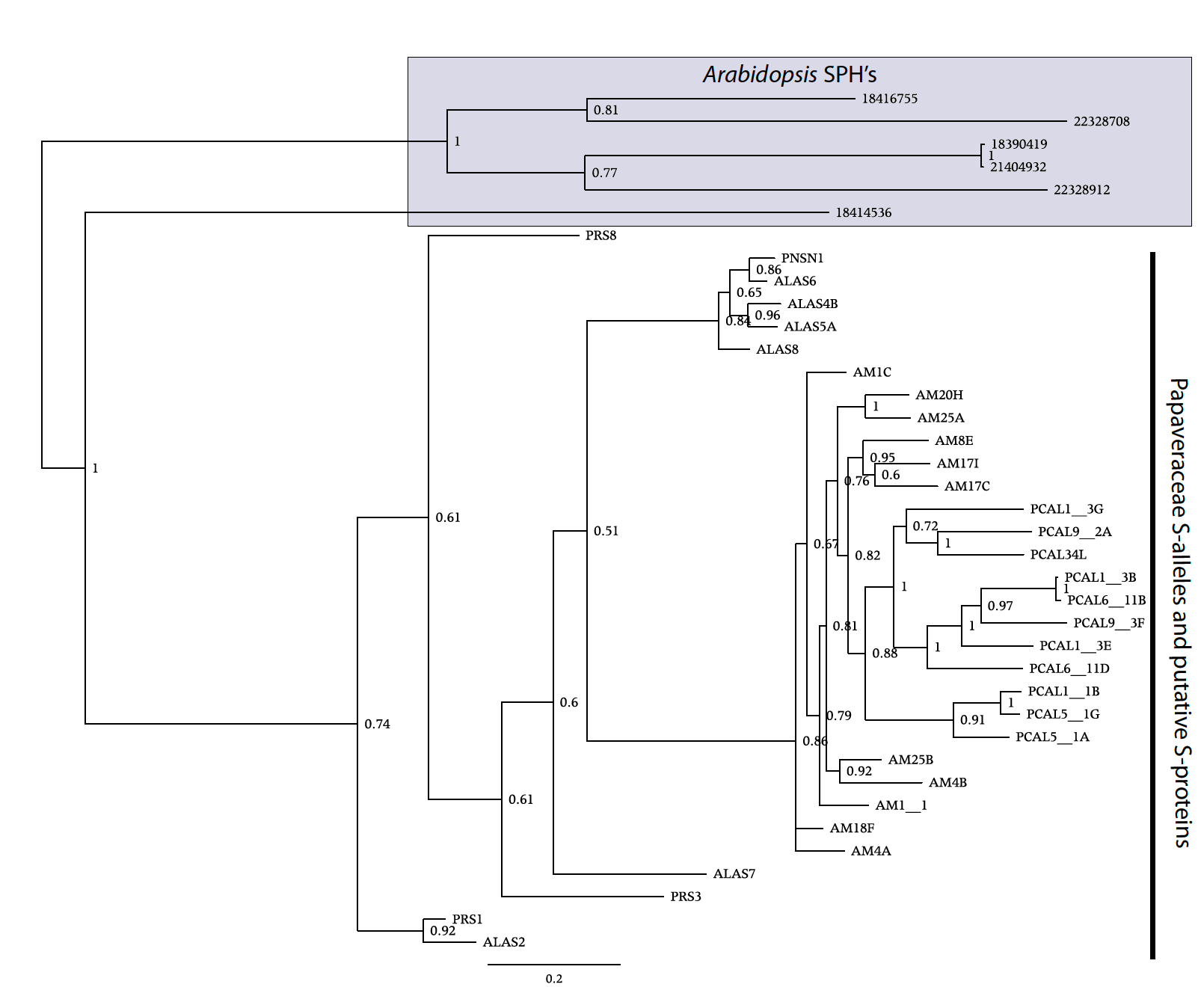


**FIGURE S1**. Bayesian phylogeny of *Papaver* S-alleles (*PrS1, PrS3, PrS8*, and *PnSn1*) and 28 putative Papaveraceae *S*-alleles showing their relationship to 6 *Arabidopsis thaliana* S-protein homologues (SPH’s). The SPH’s were chosen using a maximum E-value of 10^-4^ in tBLASTx searches against the *A. thaliana* protein database. Numbers on nodes are posterior probability scores. The phylogeny is midpoint rooted but this did not effect distances between sequences. *Argemone munita* sequence S25a queried against the Flowering Plants database resulted in E-values ranging from 9e-16 to 5e-20 with closest hits to the four *Papaver* S-alleles. Similar results are obtained using any other sequence from our sample. SPH sequences were obtained from NCBI accessions 18390419, 18414536, 18416755, 22328708, 21404932 and 22328912.
