# Supplementary material for: Evolutionary Genetics of an S-Like Polymorphism in Papaveraceae with Putative Function in Self-Incompatibility
Source: PLoS One. 2011 Aug 31;6(8):e23635. doi: 10.1371/journal.pone.0023635 (PMC3166141; doi:10.1371/journal.pone.0023635)
Supplement: Figure S2 — Coalescent simulation of the effect of sequences from a paralogous gene on recombination detection. Some putative S-alleles in our data set may belong to one or more duplicated, paralogous S-pseudogene. These are not expected to be under balancing selection and are likely to be released from recombination suppression. Therefore, even if functional S-alleles experience complete recombination suppression, contamination of the dataset by recombining S-pseudogenes could cause statistically detectable recombination. The goal of this simulation was to evaluate the effect of S-pseudogene haplotypes on the detection of recombination in a combined data set. We conducted coalescent simulations with Hudson's [77] ms program, modified to allow changes in recombination rate over time. The allelic genealogy of S-alleles can be approximated with a neutral coalescent with a scaling factor, f [78]. We assumed that the population parameter, θs, of S-alleles to be 4 f Ne μ, where Ne is the neutral effective population size and μ is a neutral mutation rate. For the paralogous pseudo-gene, which has been released from balancing selection, the population parameter was set to that of a neutral gene (θp = 4 Ne μ). At td generations ago (in units of 4 Ne f), there was a duplication of an S-allele and a paralogous pseudo-gene was created. We assumed that functional S-alleles do not recombine (the gray area), but the pseudo gene is released from recombination suppression and recombination within the paralogous alleles can occur immediately after the gene duplication (black region) with the recombination rate between the neighboring nucleotide sites of r per generation. Since we assumed that the pseudo gene was created by a single duplication event, we forced the S-pseudogene to have a severe bottleneck of θb for tb generations (in units of 4 Ne f). In the coalescent simulation, we assumed that we have sampled 24 sequences, of which some proportion, c (25%, 50%, or 75%), is actually contaminat [file pone.0023635.s002.doc]

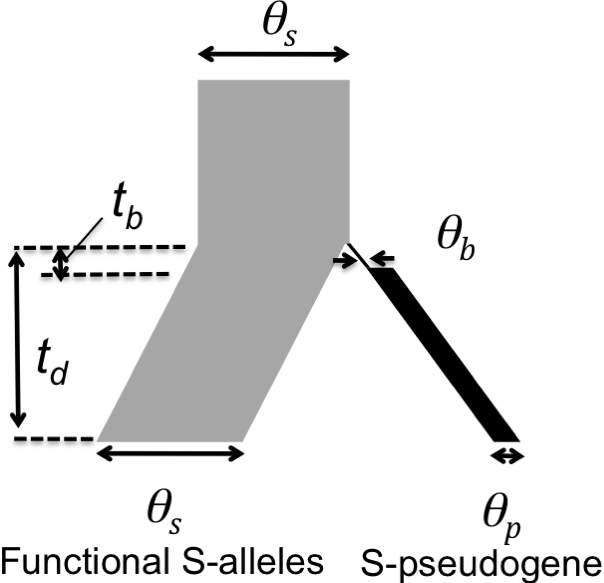


**FIGURE S2**. Coalescent simulation of the effect of sequences from a paralogous gene on recombination detection. Some putative S-alleles in our data set may belong to one or more duplicated, paralogous S-pseudogene. These are not expected to be under balancing selection and are likely to be released from recombination suppression. Therefore, even if functional *S*-alleles experience complete recombination suppression, contamination of the dataset by recombining *S*-pseudogenes could cause statistically detectable recombination. The goal of this simulation was to evaluate the effect of *S*-pseudogene haplotypes on the detection of recombination in a combined data set. We conducted coalescent simulations with Hudson’s [77] ms program, modified to allow changes in recombination rate over time. The allelic genealogy of S-alleles can be approximated with a neutral coalescent with a scaling factor, *f* [78]. We assumed that the population parameter, *θ_s_*, of *S*-alleles to be *4 f N_e_ μ*, where *N_e_* is the neutral effective population size and *μ* is a neutral mutation rate. For the paralogous pseudo-gene, which has been released from balancing selection, the population parameter was set to that of a neutral gene (*θ_p_ = 4 N_e_ μ*). At *t_d_* generations ago (in units of *4 N_e_ f*), there was a duplication of an S-allele and a paralogous pseudo-gene was created. We assumed that functional S-alleles do not recombine (the gray area), but the pseudo gene is released from recombination suppression and recombination within the paralogous alleles can occur immediately after the gene duplication (black region) with the recombination rate between the neighboring nucleotide sites of *r* per generation. Since we assumed that the pseudo gene was created by a single duplication event, we forced the S-pseudogene to have a severe bottleneck of *θ_b_* for *t_b_* generations (in units of *4 N_e_ f*). In the coalescent simulation, we assumed that we have sampled 24 sequences, of which some proportion, *c* (25%, 50%, or 75%), is actually contamination from the S-pseudogene. For each genealogical tree created by the coalescent simulation, DNA sequence evolution was simulated by Seq-Gen version 1.3.2 [79] under the Jukes-Cantor model [80], the non-parametric permutation tests of recombination were conducted with permute (r^2^ statistics) in OmegaMap v0.5 [33] and we recorded the proportion of simulations with the *p*-value ≤ 0.05. We assumed a sequence length of 327 bp, which is close to that of our data, and the following parameters were used: *f* = 20; *θ_s_* per site = 0.01, 0.1, 0.2, 0.3, 1, or 2; *t_d_* = 0.01, 0.1, 0.1, 1, 2, or 3; *r/μ* =2, 20, 60, 100, 200, or 2000; *t_b_*=0.005; *θ_b_* per site = *θ_s_* /2000000. All factorial combinations of the parameter values were used, and 1000 simulations were run for each set. In our simulations, the probability of detecting recombination with the nonparametric permutation did not exceed 0.075. This is because compared to the large diversity of S-alleles, the newly duplicated pseudo-gene has a low diversity and recombination within the pseudogene does not contribute much to the detection of recombination. This results suggest that it is unlikely that the recombination detected in our dataset is due to inclusion of S-pseudogenes
