# Supplementary material for: Evolutionary Genetics of an S-Like Polymorphism in Papaveraceae with Putative Function in Self-Incompatibility
Source: PLoS One. 2011 Aug 31;6(8):e23635. doi: 10.1371/journal.pone.0023635 (PMC3166141; doi:10.1371/journal.pone.0023635)
Supplement: Table S1 — Segregation of parental alleles among full sibs used for testing the relationship between putative SI genotype and phenotype. Tests compare expectations for two alleles from each parent segregating separately to create four offspring genotypes. For crosses involving Pl. californicus, two sequences from one parent were always found together in offspring or else neither was found. These are considered linked polymorphisms (21a,b) and (26a,b). From plant 26, an alternative allele (26c) was recovered in offspring where 26a,b was absent (see main text). (DOC) [file pone.0023635.s003.doc]

**TABLE S1**. Segregation of parental alleles among full sibs used for testing the relationship between putative SI genotype and phenotype. Tests compare expectations for two alleles from each parent segregating separately to create four offspring genotypes. For crosses involving *Pl. californicus*, two sequences from one parent were always found together in offspring or else neither was found. These are considered linked polymorphisms (21a,b) and (26a,b). From plant 26, an alternative allele (26c) was recovered in offspring where 26a,b was absent (see main text).

| Alleles from *A. munita* 4 | | Alleles from *A. munita* 25 |
| --- | --- | --- |
| 4a | 4d |  |
| 8 | 5 | 25a |
| 4 | 8 | 25b |
|  |  | χ^2^, 3df = 2.0 NS |

| Alleles from *A. munita* 1 | | Alleles from *A. munita* 8 |
| --- | --- | --- |
| 1c | 1d |  |
| 2 | 1 | 8a |
| 2 | 2 | 8b |
|  |  | Too few data |

| Alleles from *P. californicus* 1 (=34) | | Alleles from *Pl. californicus*  2=21 |
| --- | --- | --- |
| 34I | 34L |  |
| 6 | 8 | 21a,b |
| 4 | 3 | nothing |
|  |  | χ^2^, 3df = 2.0 NS |

| Alleles from *P. californicus* 1 (=34) | | Alleles from *Pl. californicus*  3=26 |
| --- | --- | --- |
| 34I | 34L |  |
| 5 | 7 | 26a,b |
| 7 | 3 | 26c |
|  |  | χ^2^, 3df = 2.0 NS |
