# Supplementary material for: Evolutionary Genetics of an S-Like Polymorphism in Papaveraceae with Putative Function in Self-Incompatibility
Source: PLoS One. 2011 Aug 31;6(8):e23635. doi: 10.1371/journal.pone.0023635 (PMC3166141; doi:10.1371/journal.pone.0023635)
Supplement: Table S2 — Estimated divergence times among taxa. Divergence times were estimated as the number of silent substitutions per site divided by the silent substitution rate (substitutions per site per year). Three genes were used for these estimates: the ribosomal internal transcribed spacer (ITS) and chloroplast genes (rbcL and atpB). Sequences were obtained from NCBI (AF057672, AF098922, AF305331, DQ912885, U86621, U86632, U86630, FJ626614, U86393, U86394, U86395, U86399), and the number of silent substitutions was estimated from DNASP [69]. Several estimates of angiosperm silent substitution rates were obtained for each gene [81]–[83], and the most extreme values were used for the min and max estimates of Papaveraceae divergence times. (DOC) [file pone.0023635.s004.doc]

**TABLE S2**. Estimated divergence times among taxa. Divergence times were estimated as the number of silent substitutions per site divided by the silent substitution rate (substitutions per site per year).  Three genes were used for these estimates:  the ribosomal internal transcribed spacer (ITS) and chloroplast genes (*rbc*L and *atp*B). Sequences were obtained from NCBI (AF057672, AF098922, AF305331, DQ912885, U86621, U86632, U86630, FJ626614, U86393, U86394, U86395, U86399), and the number of silent substitutions was estimated from DNASP [69]. Several estimates of angiosperm silent substitution rates were obtained for each gene [81-83], and the most extreme values were used for the min and max estimates of Papaveraceae divergence times.

|  |  | **Divergence time** | |
| --- | --- | --- | --- |
| **Taxa** | | **min** | **max** |
| Romneya | Argemone | 10.8 | 132.2 |
| Platystemon | Romneya | 19.8 | 171.6 |
| Platystemon | Argemone | 21.5 | 192.7 |
| Papaver | Romneya | 26.5 | 169.0 |
| Papaver | Argemone | 29.6 | 200.6 |
| Papaver | Platystemon | 41.5 | 238.9 |
