# Supplementary material for: Evolutionary Genetics of an S-Like Polymorphism in Papaveraceae with Putative Function in Self-Incompatibility
Source: PLoS One. 2011 Aug 31;6(8):e23635. doi: 10.1371/journal.pone.0023635 (PMC3166141; doi:10.1371/journal.pone.0023635)
Supplement: Table S3 — Non-parametric permutation analyses of the correlation between linkage disequilibrium and distance (top) and coalescent likelihood estimates of the population mutation (θ = 4Neμ) and recombination (ρ = 4Ner) rates with 95% highest posterior densities (bottom). (DOC) [file pone.0023635.s005.doc]

**Table S3**. Non-parametric permutation analyses of the correlation between linkage disequilibrium and distance (top) and coalescent likelihood estimates of the population mutation (θ = 4N_e_μ) and recombination (ρ = 4N_e_r) rates with 95% highest posterior densities (bottom).

|  | ***Argemone munita*** | |  | ***Platystemon californicus*** | |
| --- | --- | --- | --- | --- | --- |
|  | Correlation | *P*-value |  | Correlation | *P*-value |
| ***r*^2^** | -0.0147 | 0.129 |  | -0.0689 | 0.001 |
| ***D*'** | -0.0261 | 0.139 |  | -0.0533 | 0.014 |
| **G4** | -0.0212 | 0.202 |  | -0.0426 | 0.035 |
|  |  |  |  |  |  |
|  | Mean (95% HPD) | |  | Mean (95% HPD) | |
| **θ** | 0.94 (0.78, 1.15) | |  | 2.27 (1.93, 2.69) | |
| **ρ** | 3.01 (2.49, 3.61) | |  | 1.54 (1.23, 1.94) | |
| ***r*/μ** | 3.2 | |  | 0.68 | |
