# Supplementary material for: Evolutionary Genetics of an S-Like Polymorphism in Papaveraceae with Putative Function in Self-Incompatibility
Source: PLoS One. 2011 Aug 31;6(8):e23635. doi: 10.1371/journal.pone.0023635 (PMC3166141; doi:10.1371/journal.pone.0023635)
Supplement: Text S1 — Methods describing estimation of recombination rates in A. munita and P. californicus . (DOC) [file pone.0023635.s006.doc]

**Supplementary Methods and Results**

**Recombination estimates:** Sequences from *Pl. californicus* and *A. munita* differed relative to one another and to *Papaver* in average levels of divergence and in phylogenetic structure suggesting the possibility that recombination rates differed among taxa. We utilized population genetics and coalescent approaches to detect recombination and estimate population recombination rates of 3’ datasets (325 bp) which had the most sequences, 28 from *A. munita.* For *Pl. californicus*, seven sequences obtained using nested PCR of 3’ RT-PCR products lacked data for the 3’ end and were not used, leaving 24 sequences for this analysis. We first tested for a significant association between linkage disequilibrium and distance between sites using *permute* in OmegaMap v0.5 [33]. This method is a non-parametric permutation test of whether the correlation between linkage disequilibrium (LD) and physical distance is stronger than expected under the null hypothesis of no recombination [77]. The permutation test uses three measures of LD as follows: , when *D* ≤ 0; when ≥ 0, , and a modified *r*^2^ that uses sites that may not have all four genotypes termed ‘G4’ [78].

We then estimated the population recombination rate, ρ = 4N_e_*r* for *A. munita* and *Pl. californicus* 3’ datasets using a composite likelihood estimator in OmegaMap [34]. Because we are primarily concerned with average relative estimates of ρ between *A. munita* and *Pl. californicus*, starting prior values were set to a constant rather than variable model along the sequences using an improper inverse distribution of ρ over a range of 4N_e_*r* between 10^7^ - 10^3^. The MCMC chain was run for 500,000 generations sampling every 500^th^ generation.

We found significant evidence of a correlation between linkage disequilibrium and distance, a signature of recombination, in *Pl. californicus* but not *A. munita* (Table S3). However, the general absence of phylogenetic structure among alleles from *A. munita* (Fig. 3) suggests processes in addition to point mutations may be involved in their evolution. Gene conversion involving exchange of short tracts of nucleotides might fail to leave a pattern of decay of linkage disequilibrium by distance [58], [84-86]. Despite the lack of statistically significant evidence of a relationship between linkage disequilibrium and physical distance, the estimated population recombination rate (ρ) is significantly greater in *A. munita* than *Pl. californicus* as is the ratio of recombination rate to mutation rate (Table S3). This analysis also confirms that synonymous genetic diversity (**θ**) is significantly lower for *A. munita* than for *Pl. californicus*.

Estimates of recombination could be affected by the presence of paralogous sequences in our sample. We explored through simulation the effect that sequences from a non-functional paralogous locus would have on recombination detection. Because sequences from the paralagous locus would not have the level of polymorphism found in a locus such as *S* that is under balancing selection, they would have little effect on estimates of recombination (Figure S2).

SUPPLEMENTARY REFERENCES

84. McVean GAT, Awadalla P, Fearnhead P (2002) A coalescent-based method for detecting and estimating recombination from gene sequences. Genetics 160: 1231–1241.

85. Meunier J, Eyre-Walker A (2001) The correlation between linkage disequilibrium and distance. Implications for recombination in hominid mitochondria. Mol Biol Evol 18: 2132-2135.

86. Gay J, Myers S, McVean G (2007) Estimating meiotic gene conversion rates from population genetic data. Genetics 177: 881-894.
